# Multidimensional Recurrence Quantification Analysis (MdRQA) for the Analysis of Multidimensional Time-Series: A Software Implementation in MATLAB and Its Application to Group-Level Data in Joint Action

**DOI:** 10.3389/fpsyg.2016.01835

**Published:** 2016-11-22

**Authors:** Sebastian Wallot, Andreas Roepstorff, Dan Mønster

**Affiliations:** ^1^Max Planck Institute for Empirical AestheticsFrankfurt, Germany; ^2^Interacting Minds Centre, School of Culture and Society, Aarhus UniversityAarhus, Denmark; ^3^Department of Economics and Business Economics, Aarhus UniversityAarhus, Denmark

**Keywords:** Multidimensional Recurrence Quantification Analysis, MdRQA, multidimensional time-series, correlation, dynamics, joint action, MATLAB

## Abstract

We introduce Multidimensional Recurrence Quantification Analysis (MdRQA) as a tool to analyze multidimensional time-series data. We show how MdRQA can be used to capture the dynamics of high-dimensional signals, and how MdRQA can be used to assess coupling between two or more variables. In particular, we describe applications of the method in research on joint and collective action, as it provides a coherent analysis framework to systematically investigate dynamics at different group levels—from individual dynamics, to dyadic dynamics, up to global group-level of arbitrary size. The Appendix in Supplementary Material contains a software implementation in MATLAB to calculate MdRQA measures.

## Introduction

The interest in joint action research in the past 15 years has come with an increased interest in the temporal dimension of action (Marsh et al., 2009; Knoblich et al., 2011), which offers additional information about linguistic, motor, physiological, or neuro-physiological underpinnings of that behavior (e.g., Shockley et al., 2003; Richardson and Dale, 2005; Richardson D. C. et al., 2007; Richardson M. J. et al., 2007; Dumas et al., 2010; Konvalinka et al., 2011; Louwerse et al., 2012; Fusaroli and Tylén, 2016).

Integrating information about the temporal dimension that characterizes the interaction of multiple actors always means to apply some kind of correlational analysis, with the terms “coupling” or “synchrony” used to loosely refer to more specific patterns of correlation that can be quantified. Many techniques are available to quantify patterns of correlation, such as cross-correlational methods (e.g., Konvalinka et al., 2010), methods to detect phase-coupling (Richardson M. J. et al., 2007), or methods to detect nonlinear patterns of coupling, such as techniques based on recurrence (e.g., Shockley et al., 2003) or cross mapping (Sugihara et al., 2012). However, all of these methods primarily aim at data sets with two dependent variables (i.e., measurements taken from two participants performing a joint action task). The availability of methods that are readily applicable to the analysis of dyadic data may be one of several reasons why most joint action studies to date have been performed on the level of the dyad.

Investigation of group-level behavior has been done as well, but effectively resorting to bi-variate analyses, splitting the group behavior into all possible pairings and investigating the behavior as the average of all of its pairs. Apart from the fact that it would be desirable to quantify group-level behavior more properly (Fusaroli et al., 2014), as it might not always be the same as the average behavior of the constituting dyads, there are also practical implications on how to deal with pairwise decompositions statistically: If we have a group of three people (P1, P2, P3) that interact, and we quantify the group behavior as the average of pairwise interactions, we have to somehow deal with an insufficient number of independent degrees of freedom: Say the behaviors of P1 and P2 are positively correlated, and the behaviors of P2 and P3 are positively correlated, then the behaviors of P1 and P3 are also likely positively correlated and do not add independent information. So far, workarounds have been to either ignore this over determination in pairwise group analyses (Müller and Lindenberger, 2011), or try to work with a number random sub-samples of pairwise data points that reflect the number of actual independent degrees of freedom (e.g., Wallot et al., 2016).

The goal of the present paper is to introduce a multidimensional correlation technique, Multidimensional Recurrence Quantification Analysis (MdRQA), as a method to analyze group-level behavior of groups bigger than a dyad. In the following sections, we will describe MdRQA, explain its relation to standard Recurrence Quantification Analysis of individual time-series (RQA) and Cross-Recurrence Quantification Analysis of pairs of time-series (CRQA)—both of which have already been used to analyze dynamics of dyadic behavior (Shockley et al., 2003; Richardson and Dale, 2005; Richardson D. C. et al., 2007; Richardson M. J. et al., 2007; Konvalinka et al., 2011; Louwerse et al., 2012; Lang et al., 2016; Mønster et al., 2016a; Fusaroli and Tylén, 2016). We will also compare MdRQA to Joint Recurrence Quantification Analysis (JRQA)—another recurrence method that can be used to jointly analyze two or more time series. Then, we will show the utility of MdRQA, applying it to data from a joint action study featuring groups of three participants working on a joint production task. We show a correlation between group level dynamics of a physiological marker of arousal and independent outcome measures of the joint task. In accordance with previous analysis of the experiment using different techniques, this could not be seen at the level of aggregate individuals (Håkonsson et al., 2015) or dyads (Mønster et al., 2016a). Finally, we will end the article by discussing the interpretation of MdRQA results for group-level analysis, and summarize the advantages, disadvantages, and potential future developments of this technique. The Appendix in Supplementary Material of this paper contains MATLAB code to run the MdRQA analysis.

## Multidimensional recurrence quantification analysis (MdRQA)

MdRQA is a recurrence-based analysis technique to gauge the coordination pattern of multiple variables over time. The key concept of MdRQA, as the name suggests, is recurrence, meaning how the variables of interest repeat their values over time. MdRQA quantifies patterns of repetitions, which—depending on the interpretation of the analysis—are related to the dynamic characteristics of a multivariate system (see section “Comparison to RQA”) or characterize the coordination of a group of variables over time (see sections “Comparison to CRQA,” “Comparison to JRQA,” and “Example: Origami production task”).

**Figure 1:**
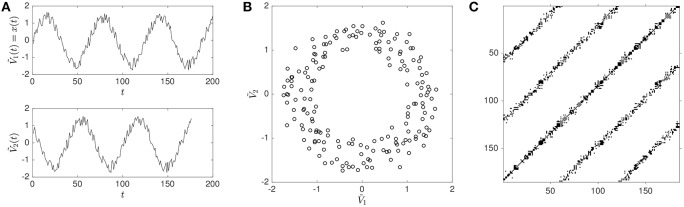
**Illustration of phase-space reconstruction and resulting RP using a noisy sine-wave**. A noisy sine-wave in the upper panel of **(A)** and its time-shifted copy (surrogate) in the lower panel of **(A)**. Reconstructed phase-space portrait **(B)**, obtained by plotting the original sine-wave ${\tilde{\text{V}}}_{1}$ against its time-delayed copy ${\tilde{\text{V}}}_{2}$. Resulting RP **(C)**, where diagonal lines of recurrences (black dots on the plot) indicate that the sine-wave signal repeats itself at intervals of roughly 60 data points. The speckled appearance of the diagonal lines indicates that repetitions are not perfect (i.e., the presence of noise).

MdRQA is a multivariate extension of simple RQA, which is an analysis technique that was developed to characterize the behavior of time-series that are the result of multiple interdependent variables, potentially exhibiting nonlinear behavior over time (Webber and Zbilut, 1994; Marwan et al., 2002). The basis of the RQA approach is phase-space reconstruction through time-delayed embedding. A phase-space is a space in which all possible states of a system under study can be charted. If full determination of the state of the system requires *D* independent variables, then the phase space has *D* dimensions. The method of time-delayed embedding allows the reconstruction of phase-space profiles from a single, one-dimensional observable, following the logic of Takens' theorem (Takens, 1981). Takens showed that if a system of interest is comprised of multiple interdependent variables that drive its dynamics (i.e., its dynamics are multidimensional), and one has access only to a single observable *x* from the system (i.e., measuring one of its dimensions), then the multidimensional dynamics of that system can be reconstructed from the single measured dimension by plotting the observable *x* against itself a certain number of times at a certain time delay (see Figure 1). The starting point for the method is the measured values of the variable *x*:

(1) $$\begin{array}{l} \text{x}=\left(x_{1},x_{2},x_{3},\ldots ,x_{n}\right) \end{array}$$

where **x** is a vector with values *x*_1_ to *x*_*n*_ representing the time-series of the variable *x* sampled at regular times *t*_1_, *t*_1_ + Δ*t*, *t*_1_ + 2Δ*t*, … *t*_1_ + (*n* − 1)Δ*t*. If we know (or can estimate) the true dimension *D* of the dynamical system from which we have sampled *x* then we can construct *D*-dimensional vectors of the form:

(2) $$\begin{array}{l} {\text{V}}_{1}=\left(x_{1},x_{1+\tau },x_{1+2\tau },\ldots ,x_{1+\left(D-1\right)\tau }\right) \end{array}$$

Note that the elements of **V**_1_ are all elements from **x**, starting with *x*_1_ sampled at time *t*_1_ and then using values at later times, such as *x*_1 + τ_ sampled at *t*_1_ + τΔ*t*. Since the later times are all delayed relative to *t*_1_ by an integer multiple of τΔ*t*, the constant τ is called the time-lag. We can construct a similar vector **V**_2_ by starting with *x*_2_ sampled at *t*_2_ = *t*_1_ + Δ*t*, and in fact we can construct *n* − (*D* − 1)τ such vectors, that can be arranged in a matrix:

(3) $$\begin{array}{l} \text{V}=\left(\begin{array}{c} {\text{V}}_{1}\\ {\text{V}}_{2}\\ \vdots \\ {\text{V}}_{n-\left(D-1\right)\tau } \end{array}\right)=\left(\begin{array}{cccc} x_{1} & x_{1+\tau } & \ldots & x_{1+\left(D-1\right)\tau }\\ x_{2} & x_{2+\tau } & \ldots & x_{2+\left(D-1\right)\tau }\\ \vdots & \vdots & \; & \vdots \\ x_{n-\left(D-1\right)\tau } & x_{n-\left(D-2\right)\tau } & \ldots & x_{n} \end{array}\right) \end{array}$$

Note that the rows are the *D* -dimensional phase-space vectors that we set out to construct above, while the columns are time-delayed versions of the first *n* − (*D* − 1)τ elements of the vector **x**, delayed by 0τ, 1τ, 2τ, etc. The row index is a measure of time and each column index corresponds to a dimension in phase-space. Thus, the row vectors **V**_*i*_ constitute points in the phase-space portrait of the multidimensional dynamics of the system from which the observable *x* was taken. The column vectors, ${\tilde{\text{V}}}_{j}$, *j* = 1, 2, … *D* are time series vectors, corresponding to the reconstructed dimensions of the phase space, and in particular ${\tilde{\text{V}}}_{1}$ is the measured variable *x* from which the other dimensions are constructed. RQA is a method to statistically describe these multidimensional dynamics through the concept of recurrence in phase-space. RQA statistics are based on the recurrence plot (RP), which was invented as a means to graphically display the dynamics of a multidimensional phase-space (Eckmann et al., 1987). In essence, the RP describes repetitions of the values of **V** in its phase-space. A point RP_*ij*_ in the RP is considered recurrent if the distance ||**V**_*i*_(**x**) − **V**_*j*_(**x**)|| between the point **V**_*i*_(**x**) (at time *t*_*i*_) and the point **V**_*j*_(**x**) (at time *t*_*j*_) is smaller than the threshold *T*. This can be written as

(4) $$\begin{array}{l} \text{R}{\text{P}}_{ij}=\Theta \left(T-||{\text{V}}_{i}\left(\text{x}\right)-{\text{V}}_{j}\left(\text{x}\right)||\right), \end{array}$$

where Θ(*x*) is the Heaviside step function, which has the value 0 for *x* < 0 and 1 for *x* ≥ 0. Throughout the remainder of the manuscript, values of the threshold parameter *T* are relative to a Euclidean distance norm of the respective phase-spaces.

As an example, imagine that we want to measure the position of a person on a merry-go-round, then assuming that the person does not move up and down, we only need two variables *x* and *y* to determine the position of the person at a given time. These two variables make up the phase-space of the system^1^. If we only measured one of these variables, say *x*, then we can reconstruct the full phase space from this variable alone using the method described above. Figure 1 illustrates the process where the measured values of *x* have been simulated by using a sine-wave with added noise.

**Figure 2:**
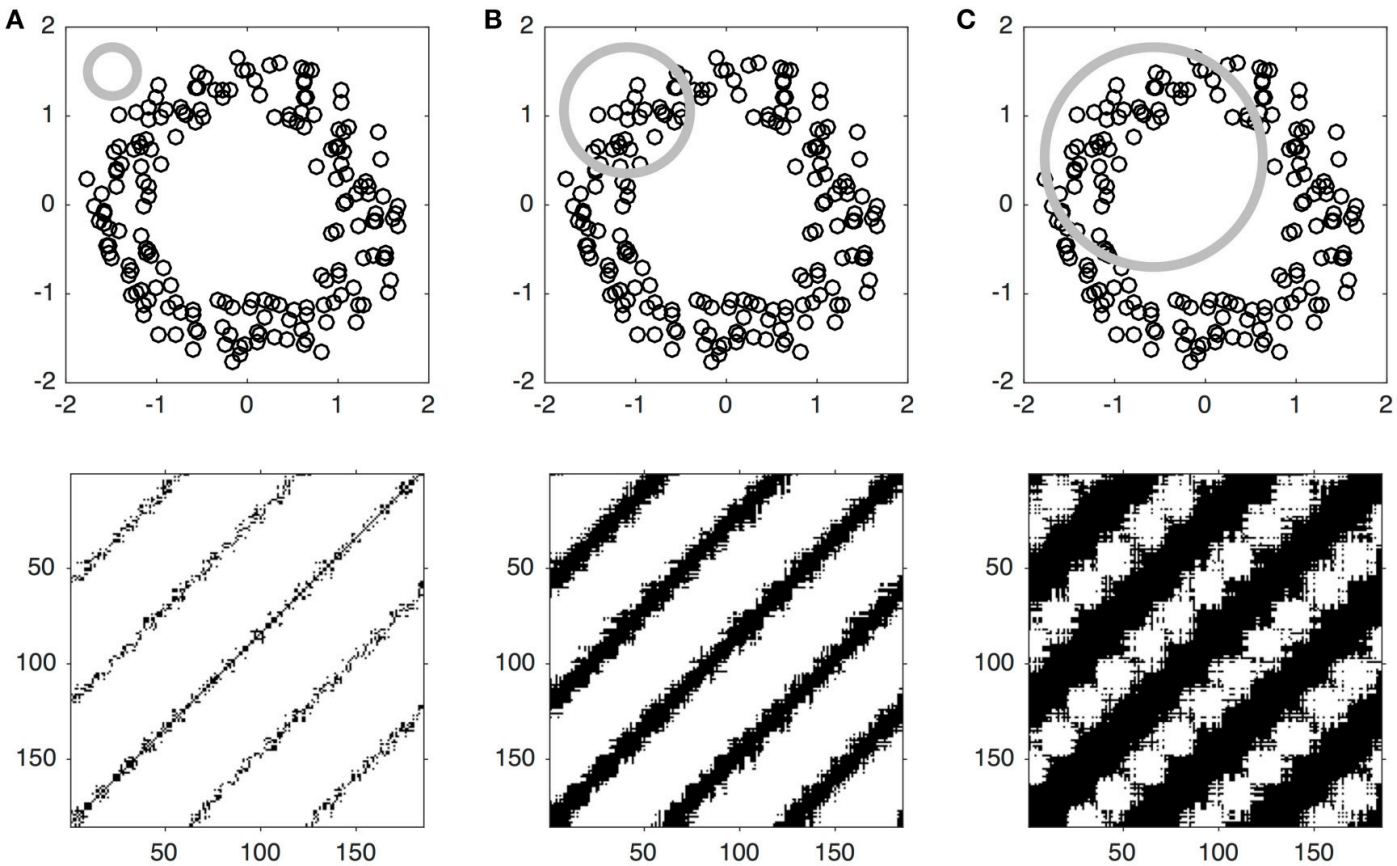
**Illustration of the effect of the threshold parameter on the percentage of recurrence points in an RP**. The upper panels **(A–C)** show the same phase-spaces as in Figure 1, but with an application of increasingly larger threshold, within which points in phase-space are counted as being recurrent, illustrated by a gray circle. The lower panels **(A–C)** show that the corresponding RP yield increasingly higher percentages of recurrence points, evident by the increasing thickness of the diagonal line patterns on the plots.

Because repetitions are usually never exact, either due to intrinsic fluctuations of the system's dynamics or measurement noise, a threshold parameter *T* is applied, within which values in phase-space are counted as being recurrent or not (see Figure 2).

MdRQA extends RQA by allowing the use of additional *measured* variables from the system under study to be used as dimensions in phase-space. Hence, instead of quantifying the dynamics of a *D* -dimensional system from a single observable *x* by using the *D* -dimensional vectors **V**_*i*_(*x*), MdRQA allows us to quantify the dynamics by using a number *N* of observables *y*_1_, *y*_2_, … *Y*_*N*_ to construct the phase-space:

(5) $$\begin{array}{l} \text{W}=\left(\begin{array}{c} {\text{W}}_{1}\\ {\text{W}}_{2}\\ \vdots \\ {\text{W}}_{n} \end{array}\right)=\left(\begin{array}{cccc} y_{1,1} & y_{2,1} & \ldots & y_{N,1}\\ y_{1,2} & y_{2,2} & \ldots & y_{N,2}\\ \vdots & \vdots & \; & \vdots \\ y_{1,n} & y_{2,n} & \ldots & y_{N,n} \end{array}\right) \end{array}$$

where **W**_*i*_ is the *N* -dimensional vector consisting of the *N* observables measured from the system sampled at time *t*_*i*_. The elements of the matrix **W** are thus given by *W*_*ij*_ = *y*_*j, i*_, where *y*_*j, i*_ is the value of *y*_*j*_ at time *t*_*i*_.

MdRQA shares commonalities with Self-Similarity Matrices (SSM): Both methods rely on the computation of a distance matrix, where distances between sequences of positions of a multidimensional array are charted. However, while SSMs operate on the Euclidean distance of this distance matrix (e.g., Junejo et al., 2008), MdRQA proceeds by operating on the thresholded distance matrix (see RP illustration in Figure 2) in order quantify the matrix in terms of the standard recurrence measures (Webber and Zbilut, 1994; Marwan et al., 2002).

Earlier attempts to use RQA on multidimensional signals were made by computing the Euclidean distance of multiple signals and analyzing the resulting distance vector, for example by Thomasson et al. (2002) (cited in Webber and Zbilut, 2005) who quantified scaling characteristics in EEG-activity as a global brain-dynamics analysis. Applying RQA directly on multidimensional signals has been done in prior studies on the analysis of joint action by (Mitkidis et al., 2015; Wallot et al., 2016) to quantify the joint dynamics of hand movement in a joint car-model building task, taking each of the four hand acceleration time-series of the collaborating builders as variables.

## Comparison to RQA

**Figure 3:**
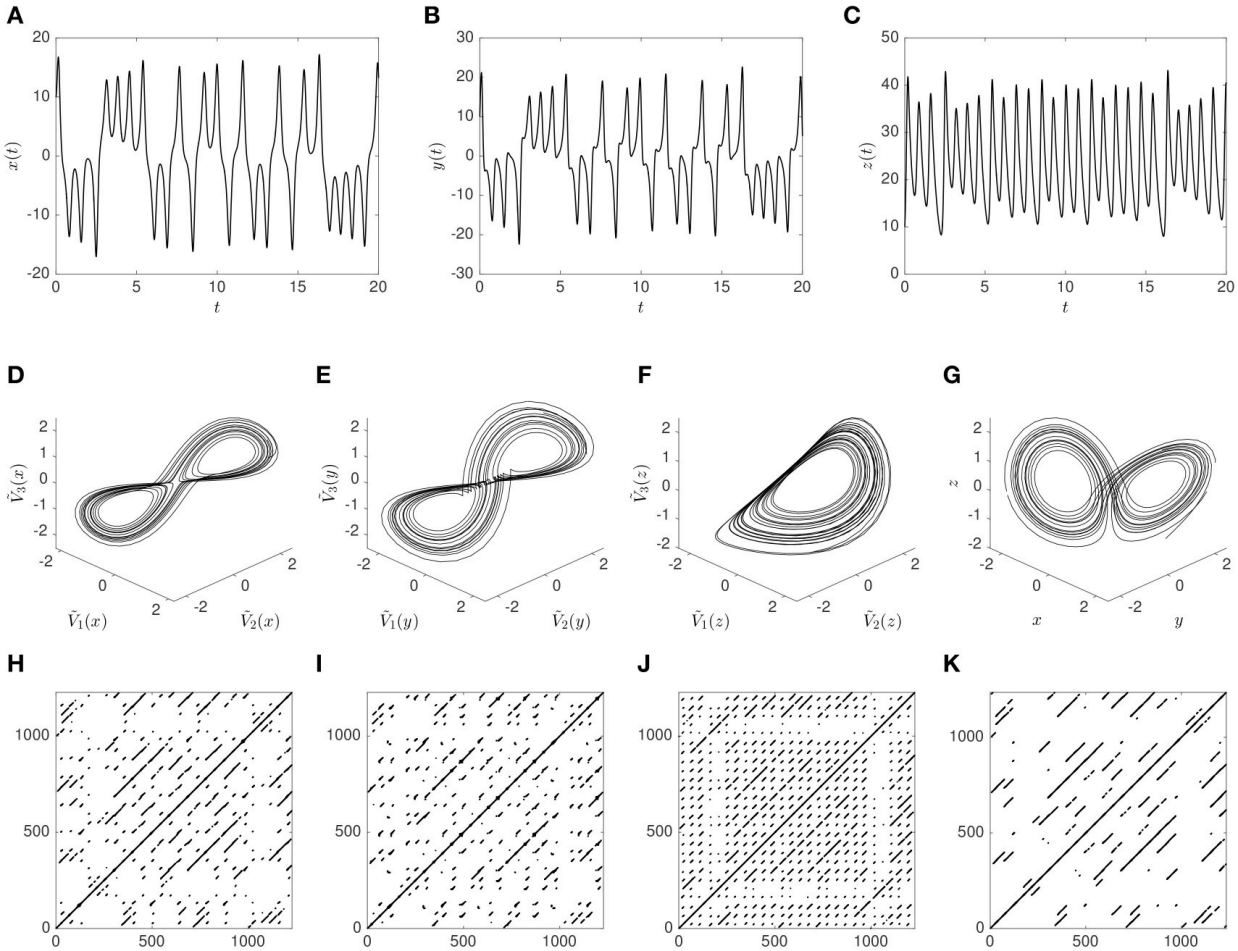
**The time series for ***x*** (A)**, *y* **(B)**, and *z* **(C)** obtained by numerical integration in the solution interval. The Lorenz attractor **(G)** is the phase space plot of *x*, *y*, and *z* shown in *z* -scored dimensions. The reconstructed attractor based on time delayed embedding of *x*, *y*, and *z* respectively, with *D* = 3 and τ = 4, is shown in **(D–F)** (also in *z* -scored dimensions). Finally recurrence plots, using a threshold *T* = 0.1 to define a recurrence, are shown for the reconstructed attractors, based on *x* **(H)**, *y* **(I)**, and *z* **(J)**; as well as based on the original attractor **(K)** with a threshold *T* = 0.08.

The relation between RQA and MdRQA has already been described above. Nevertheless, we want to illustrate how RQA can be used to infer the multidimensional dynamics of a system from a single observable, and compare this to how MdRQA allows the quantification of those dynamics by taking into account multiple observables. As an example, we choose the Lorenz system (Lorenz, 1963), a dynamic system of three coupled differential equations:

(6) $$\begin{array}{r} \frac{dx}{dt}=\sigma \left(y-x\right)\\ \frac{dy}{dt}=x\left(\rho -z\right)-y\\ \frac{dz}{dt}=xy-\beta z \end{array}$$

where the parameters σ, ρ, β are constants with positive values. In the following we have chosen the fixed values σ = 10, ρ = 28, and β = 8/3. We solve the equations numerically in the interval 0 ≤ *t* ≤ 20, giving us solutions for *x*(*t*), *y*(*t*), and *z*(*t*), shown in Figures 3A–C. The maximum time (*t* = 20) is a somewhat arbitrary choice, that was chosen simply to give enough data points to use for recurrence analysis. We resample the data from the numerical integration to ensure that all three time series *x*(*t*), *y*(*t*), *z*(*t*) are sampled uniformly with the same time values, using a sampling interval Δ*t* = 0.0162. In order to get comparable phase spaces, we further normalize the sampled time series for *x*, *y*, and *z* by using z-scores. If we plot the (z-scored) points (*x*(*t*), *y*(*t*), *z*(*t*)) for all values of *t*, we get the well-known Lorenz attractor, shown in Figure 3G. This plot shows the dynamics of the system in phase space, where the time, *t*, is no longer plotted along one of the axes, but each data point with regard to its position in the 3D space was sequentially plotted with temporal ordering on *t*.

Using the method of time-delayed embedding, we can take each of the individual dimensions, *x*, *y*, and *z*, to reconstruct the three-dimensional dynamics of the system via time-delayed embedding. The attractors, reconstructed with embedding dimension *D* = 3 and time delay τ = 4, are shown in Figures 3D–F. For the reconstructed attractors the points plotted are the row vectors **V**_1_, **V**_2_, and **V**_3_, that are created from the time-delayed values of *x*, *y*, and *z*, respectively.

The points that make up these reconstructed attractors using the time delayed embedding can be used to produce recurrence plots as shown in Figures 3H–J, by applying RQA. Note that the axes on the RPs refer to vector index, rather than time, and correspond to the full time series shown in Figures 3A–C (there are 1234 samples, and 1234 · Δ*t* = 20). Analogously, the information in all three dimensions can be used to produce the RP shown in Figure 3K, by applying MdRQA.

The figure illustrates that the time delayed embedding method relying on Takens' theorem does indeed produce reconstructed attractors (Figures 3D–F) that are isomorphic to the true attractor (Figure 3G), but it is also clear that the fidelity is not the same for all dimensions, e.g., the reconstruction based on *z*(*t*) does not properly reproduce the double-lobed structure of the original attractor. The RPs in Figures 3H–J that are based on a single variable *x*, *y*, or *z* clearly resemble each other, and also resemble the RP based on all three variables (Figure 3K). Many of the diagonal line structures are reproduced in all of the RPs, but with “noise” in the form of broken diagonal lines and points that are not part of diagonal lines seen in the RPs based on a single variable (Figures 3H–J) when compared to the RP based on all three variables (Figure 3K).

As mentioned above, the RP is not just a means to visually display the dynamics, but also allows to quantify them. Webber and Zbilut (1994) defined the first four recurrence measures, recurrence rate (RR), determinism (DET), average diagonal line length (ADL), and longest diagonal line length (LDL). These four measures quantify different aspects about the dynamics and their definitions are given in Table 1. Recurrence rate and determinism are commonly reported both as a fraction and in percent (% recurrence and % determinism).

**Table 1:** **Description of the four RQA measures RR, DET, ADL, and LDL**.

**Measure**	**Name**	**Definition**
RR	Recurrence rate	Sum of recurrent points in RP/Size of RP
DET	Determinism	Sum of diagonally adjacent recurrent points/Sum of recurrent points in RP
ADL	Average diagonal line length	Average length of diagonal lines in RP
LDL	Length of longest diagonal line	Length of longest diagonal line in RP

Further measures have been developed and are currently in development (e.g., Marwan et al., 2002). However, for the purpose of describing MdRQA as a method we will only focus on those four. Values of the four recurrence measures for the recurrence plots shown in Figures 3H–K are shown in Table 2.

**Table 2:** **Values of the RQA measures RR, DET, ADL, and LDL for the recurrence plots shown in Figures 3H–K with embedding dimension ***D*** = 3, time delay τ = 4, and threshold ***T*** = 0.01 for RQA and ***T*** = 0.008 for MdRQA)**.

	**RQA**(*x*)	**RQA**(*y*)	**RQA**(*z*)	**MdRQA**
RR (%)	0.69	0.84	0.68	0.69
DET (%)	99.4	97.4	99.5	99.9
ADL	9.12	7.84	10.3	16.4
LDL	131	118	82	167

The measures in Table 2 are consistent with the qualitative interpretation of the recurrence plots, presented above, and we also get some information that is difficult to read off a plot, e.g., that the recurrence rate is almost exactly the same in all of the RPs (with the RP based on *y* being slightly denser). The main difference between the MdRQA measures and the RQA measures is that the diagonal line structures are consistently longer in MdRQA than in RQA. This is because, in this case, MdRQA captures the true dynamics of the system, since we have all the dimensions included, whereas RQA is based on an approximation using only one of these. Moreover, this allows for comparisons of how well the individual dimensions from which the phase-spaces were reconstructed approach the original: For example, comparing the RQA values in Table 2, it seems that the dimension *x* of the Lorenz system (Figure 3A) provides a better reconstruction than *y* and particularly *z* (Figures 3B,C, respectively).

## Comparison to CRQA

Cross-Recurrence Quantification Analysis (CRQA) was probably the first multivariate extension of RQA, allowing for the analysis of two variables and their cross-recurrences (Marwan and Kurths, 2002). Besides explicitly incorporating more than one variable for analysis, CRQA also enables capturing the *relation* between the two variables, as CRQA-measures are not derived from the distances within a single phase-space profile, but are based on the distances between two profiles in phase-space. This is made explicit by comparing the formula for the recurrence plot (RP) with the formula for the cross recurrence plot (CRP). The recurrence plot is a plot of all non-zero elements of the recurrence matrix RP_*ij*_ (see Equation 4), just as the cross-recurrence plot is a plot of all non-zero elements of the cross-recurrence matrix CRP_*ij*_:

(7) $$\begin{array}{l} \text{CR}{\text{P}}_{ij}=\Theta \left(T-||{\text{V}}_{i}\left(\text{x}\right)-{\text{V}}_{j}\left(\text{y}\right)||\right) \end{array}$$

Here, as in Equation (4), *T* is the threshold parameter that determines how close two points must be to each other to count as a recurrence. The formula for the RP (Equation 4) contains the distance, ||**V**_*i*_(**x**) − **V**_*j*_(**x**)||, between two points, **V**_*i*_ and **V**_*j*_ in the reconstructed phase-space based on the points in the time series **x**, whereas the formula for the CRP contains the distance between a point **V**_*i*_(**x**) in the phase space reconstructed with points from **x** and a point **V**_*j*_(**y**) reconstructed with points from **y**.

As a model system to compare MdRQA with CRQA we choose a system of two coupled van der Pol oscillators, whose dynamics are governed by the coupled, second-order, differential equations:

(8) $$\begin{array}{r} \frac{d^{2}x}{dt^{2}}=\mu \left(1-x^{2}\right)\frac{dx}{dt}-x+\epsilon_{1}\left(x-y\right)\\ \frac{d^{2}y}{dt^{2}}=\mu \left(1-y^{2}\right)\frac{dy}{dt}-y+\epsilon_{2}\left(y-x\right) \end{array}$$

**Figure 4:**
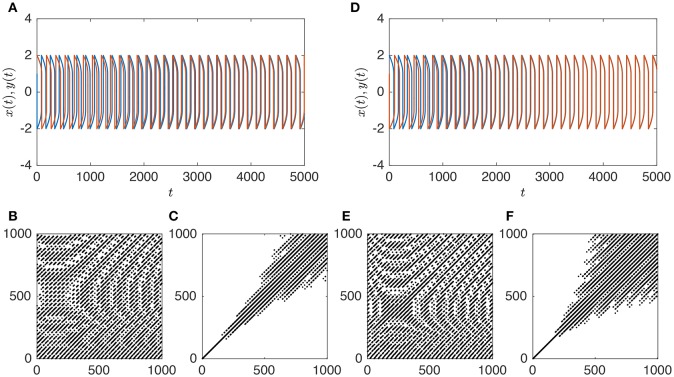
**Temporal dynamics (A)** of a system of two (in red and blue) coupled van der Pol oscillators with ϵ_1_ = 0.01. CRP **(B)** and MdRP **(C)** for the time series shown in **(A)** with *D* = 2, τ = 1, and *T* = 0.01 for both CRP and MdRP. **(D–F)** show the same, but for ϵ_1_ = 0.02. In both cases ϵ_2_ = 5ϵ_1_.

We fix μ = 100 and choose an asymmetric coupling between the variables, so that ϵ_2_ = 5ϵ_1_, leaving only one free parameter in the system. A Cross-Recurrence Plot (CRP) and Multidimensional Recurrence Plot (MdRP) for the coupled van der Pol oscillators are shown in Figure 4 for two different values of the coupling.

Comparing the time series at low coupling (Figure 4A) with the time series at high coupling (Figure 4D) it is evident that the two oscillators synchronize and become phase-locked for the high value of the coupling, whereas this happens on a longer time scale for low coupling. Here we are interested in whether CRQA and MdRQA capture this difference. There is a clear difference between the RPs produced by CRQA and MdRQA, both at low (Figures 4B,C) and high (Figures 4E,F) coupling. However, the RPs for CRQA at both low (Figure 4B) and high (Figure 4E) coupling look qualitatively similar, as do the RPs for MdRQA (Figures 4C,F). The RPs for MdRQA are indicative of a system that is initially non-periodic, but switches to periodic behavior. The RPs based in CRQA are somewhat insensitive to this, because the CRQA method is based on recurrence between to different phase-space trajectories—one built from *x* and one built from *y* —and these are both individually periodic, which masks the initial non-periodicity of the combined system.

**Figure 5:**
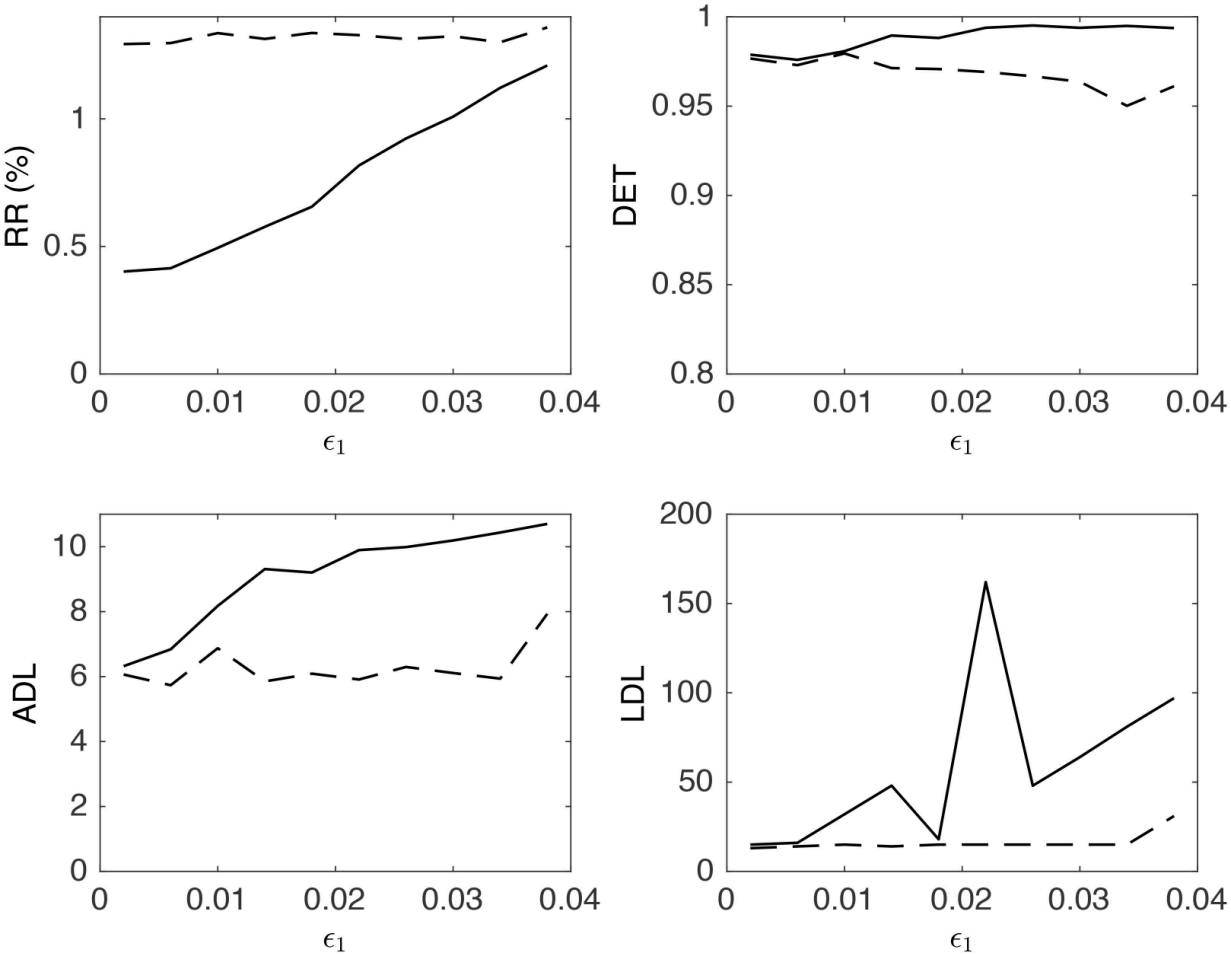
**Recurrence measures RR, DET, ADL, and LDL for CRQA (dashed lines) and MdRQA (solid lines) as a function of the coupling constant ϵ_**1**_**.

To investigate the difference between CRQA and MdRQA in this example, we show in Figure 5 how the recurrence measures obtained from the (cross-)recurrence plots vary as a function of coupling strength ϵ_1_. This figure demonstrates, quantitatively, that both methods are sensitive to changes in coupling. However, the MdRQA-based measures exhibit stronger, and more convergent correlations with coupling strength, which is evident from the correlation coefficients in Table 3: The MdRQA measures have generally high correlations with ϵ_1_, compared to the lower (in one case even negative) correlations between ϵ_1_ and the CRQA measures.

**Table 3:** **Pairwise Pearson correlation coefficients between the RQA measures shown in Figure 5 and the coupling constant ϵ_**1**_**.

	**RR**			**DET**		
	ϵ_1_	CRQA	MdRQA	ϵ_1_	CRQA	MdRQA
ϵ_1_	–	0.48	0.99	–	−0.86	0.89
CRQA		–	0.42		–	−0.74
MdRQA			–			–
	**ADL**			**LDL**		
	ϵ_1_	CRQA	MdRQA	ϵ_1_	CRQA	MdRQA
ϵ_1_	–	0.43	0.94	–	0.60	0.60
CRQA		–	0.32		–	0.35
MdRQA			–			–

This example of two coupled van der Pol oscillators illustrates the utility of MdRQA in detecting the coupling between two systems. It is important to note that this does not generally imply a greater sensitivity of MdRQA relative to CRQA, as we have not systematically tested different systems and their coupling properties.

## Comparison to JRQA

Another extension of the basic recurrence plot is the Joint Recurrence Plot (JRP), which also allows investigations of the relation between multiple variables (see Marwan et al., 2007, for an introduction to JRPs and comparisons between JRPs and CRPs). While CRPs capture the commonalities between two signals as the distance between their phase-space profiles (see section above), JRPs capture the commonalities between two signals as coinciding instances of recurrence between the individual RPs of those signals. So first, proper RPs are constructed for each signal, and then their JRP can simply be computed by joining the plots together, so that common instances of recurrences are kept, but instances of recurrence that are different between the two plots are discarded. In the formula for the JRP, this is achieved as a product of two Heaviside functions, which is 1 if they are both 1 (recurrence in both variables) and 0 otherwise.

(9) $$\begin{array}{l} \text{JR}{\text{P}}_{ij}=\Theta \left(T_{x}-||{\text{V}}_{i}\left(\text{x}\right)-{\text{V}}_{j}\left(\text{x}\right)||\right)\cdot \Theta \left(T_{y}-||{\text{V}}_{i}\left(\text{y}\right)-{\text{V}}_{j}\left(\text{y}\right)||\right) \end{array}$$

Here, we allow for different thresholds *T*_*x*_ and *T*_*y*_ in the two phase spaces.

This plot can then be quantified just as a regular recurrence plot, yielding a Joint Recurrence Quantification Analysis (JRQA). Moreover, Marwan et al. (2007) also proposed a multivariate extension for JRQA, where the JRP is computed not just by joining two, but arbitrarily many individual RPs, based on a number (*d*) of observed variables *y*_1_, *y*_2_ … *y*_*d*_:

(10) $$\begin{array}{l} \text{JR}{\text{P}}_{ij}=\overset{d}{\underset{k=1}{\prod }}\Theta \left(T_{k}-||{\text{V}}_{i}\left({\text{y}}_{k}\right)-{\text{V}}_{j}\left({\text{y}}_{k}\right)||\right) \end{array}$$

Hence, similar to MdRPs, JRPs also offer a way to quantify the simultaneous dynamics of more than two variables. The difference is that MdRPs are based on a phase-space that incorporate the component signals, JRPs are based on the RPs of the individual component signals which are joint together. In other words, MdRQA quantifies the commonalities based on the recurrence profile of a multi-component-signal phase-space, while multivariate JRPs quantify the commonalities based the recurrence profiles of multiple individual component signals. Using the Lorenz-system, we can illustrate the similarities and differences of how multivariate JRPs and MdRPs handle multivariate time series.

Table 4 summarizes the quantitative differences between the multivariate JRP and the MdRP of the Lorenz system: In general, the values are of comparable magnitude, except for RR which is a factor 6 smaller for JRP compared to MdRP. This is due to the fact that the structure on the JRP is contingent on recurrence in all the three constituent RPs simultaneously. Since joint recurrence will not be perfect across the plots, many of the recurrent instances in the constituent plots will disappear in the JRP because recurrence is absent in at least one of the other RPs.

**Table 4:** **Values of the RQA measures RR, DET, ADL, and LDL for multivariate JRP shown in Figure 6A, and the MdRP shown in Figure 6/Figure 3K**.

	**JRP**	**MdRP**
RR (%)	0.14	0.84
DET (%)	98.1	97.4
ADL	11.9	7.84
LDL	82	118

## Example: origami production task

As we have shown in the examples above, MdRQA can be used to quantify the dynamics of a multidimensional system at different levels of description by combining information from multiple variables, and it can be used to infer the shared dynamics of multiple time-series, similarly to CRQA or JRQA. In the following, we will apply MdRQA to empirical data to demonstrate how it can be used to systematically analyze group dynamics at different levels of aggregation: individuals, dyads, and at a global group level. In order to do so, we present a re-analysis of a sub-set of data from a study on teamwork investigating the role of team emotions for cooperation (Håkonsson et al., 2015; Mønster et al., 2016a).

In this study, teams of three participants were asked to build origami boats together over five consecutive sessions. The participants were told that the team that built the most boats would win an extra cash prize. Participants were fitted with heart rate, skin conductance, and facial electromyography monitors to investigate the role of dynamics of emotions during teamwork. Participants were then shown how to build the boats and subsequently built as many boats as they could during three 4-min sessions. After session three, participants were shown an alternative building technique and could choose to either adopt the new technique in sessions four and/or five, or stick with the original folding technique (see Mønster et al., 2016a, for further details on the study).

While the study by Håkonsson et al. (2015) looked at static effects of emotional measures, aggregating individual team members' physiological reactions to an average score, the study by Mønster et al. (2016a) re-examined the data using CRQA to look at shared emotional dynamics between pairs of team-members. The individual physiological responses averaged at the group level showed only a marginal effect of emotions on outcomes in this team task (Håkonsson et al., 2015). However, shared emotional dynamics at the level of dyads as measured by skin conductance and electromyography of the zygomaticus major (“smiling muscle”) were influenced by task conditions (Mønster et al., 2016a). Moreover, these dynamics were predictive of subjective self-reports of the team members, as well as the decision of whether to adopt a new work routine or not.

Comparing the results of these two studies demonstrates that the dynamics of physiological markers of arousal and emotions may contain information about interpersonal decisions and subjective states, and, importantly, that aggregate shared dyadic dynamics provides different information than aggregate individual scores. However, as discussed above, dyadic analysis only paints a partial picture of the global dynamics in groups bigger than two as it is effectively an aggregate of sub-groups at an intermediate level. In the following we demonstrate that MdRQA can be used to systematically investigate different levels of dynamics, starting from the individual to dyadic (triadic, etc.) relationships within a group, up to the highest level of global group-level-dynamics.

To illustrate this, we explore one of the observables from the origami-study, the skin conductance measure. Recall, that participants were put together in groups of three with the goal of producing as many origami boats during each session as possible. However, neither the individual measures of the group processes (Håkonsson et al., 2015), nor the dyadic shared dynamics investigated using CRQA (Mønster et al., 2016a) showed any predictive relationship to the performance outcome in terms of number of boats successfully built. Of course, it could simply be the case that the observables used in this study (skin-conductance, heart-rate, electromyography of facial muscles) were not related to this aspect of group performance. However, it could also be the case that the group dynamics were not quantified at the level at which emotion-related team dynamics were relevant for team performance.

We used MdRQA to differentiate between these explanations. To that end, we subjected the individual skin-conductance records of team members to MdRQA1 and averaged the resulting measures across the team to capture the effect of the average individual skin-conductance dynamics. We denote the number *n* of measured observables taken as dimensions in MdRQA by an index number: Hence, MdRQA1 means that MdRQA was performed on a single, one-dimensional observable (equaling simple RQA), MdRQA2 means that MdRQA was performed on two, one-dimensional observables, and MdRQA*N* means that MdRQA was performed on *N*, one-dimensional observables. However, *N* does not necessarily equal the number of phase-space dimensions *D*, as time-delayed embedding is performed (see Section “A note on parameter estimation using MdRQA”).

This allowed us to explore higher-level group-dynamics as well as the individual dynamics (i.e., MdRQA1). For the dyadic level, we subjected paired skin-conductance records within each team to MdRQA2 and averaged across the three resulting pairings per team to capture the effect of dyadic dynamics within the team. To capture the global effect of group level dynamics we subjected the three skin-conductance records simultaneously to MdRQA3.

We used the following embedding parameters to perform the analysis: Delay τ = 6, embedding dimension *D* = 6 (i.e., a 3-dimensional signal embedded once, 3 · 2 = 6), threshold *T* = 0.12, using a Euclidean norm. Note that normalization of the phase-space is important to compare different signals or samples with regard to their dynamics (see Shockley et al., 2003), and various norms can be used to achieve this (Webber and Zbilut, 2005). However, the most important thing about selecting a norm parameter is to keep it constant across all data sets.

**Figure 6:**
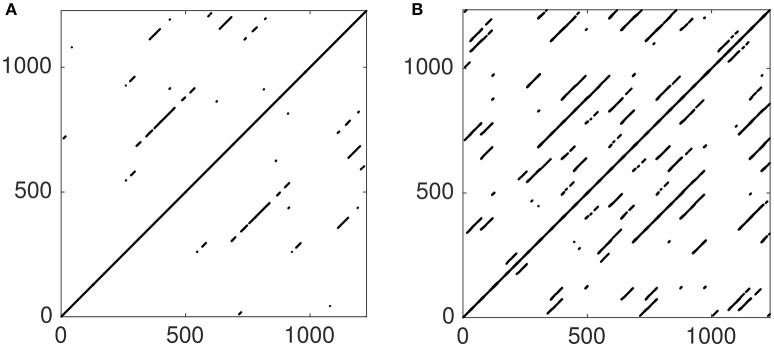
**Multivariate JPR obtained by joining the individual RPs from Figures 3H–J (A)**. MdRP from Figure 3K (B). The plots convey a similar qualitative picture of the dynamics of the Lorenz system, with the main difference that the JRP has fewer points and fewer diagonal structures than the MdRP.

**Figure 7:**
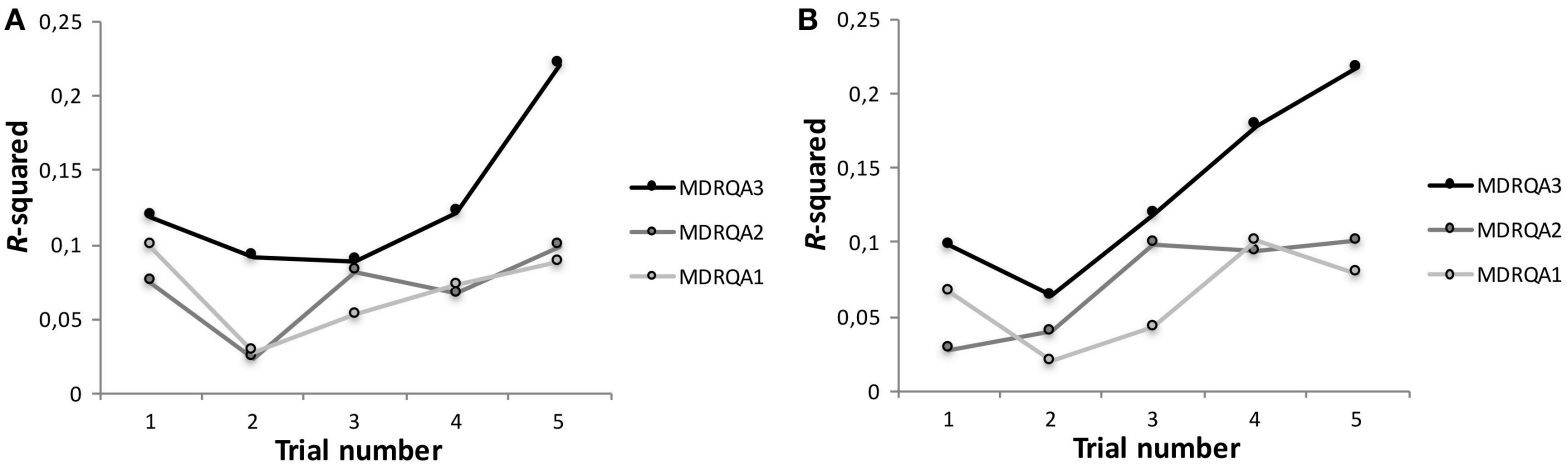
*****R***^**2**^ of a simple regression model using RR, DET, ADL, and LDL as predictors for (A)** the number of successfully built origami boats and **(B)** the number of unsuccessful attempts during each of the five trials for average individual dynamics (MdRQA1), average dyadic dynamics (MdRQA2), and group-level dynamics (MdRQA3). As all regression models had the same number of degrees of freedom (predictor *DF* = 4, residual *DF* = 95), a significant model at α = 0.05 had to explain at least 9.6% of variance (*R*^2^ = 0.096), i.e., all models with *R*^2^ > 0.096 are significant at *p* < 0.05.

Just as in the study by Mønster et al. (2016a), we computed the recurrence measures RR, DET, ADL, and LDL to capture the individual and shared skin-conductance dynamics (Table 1 described these measures). We use these four resulting MdRQA measures for average individual-level team dynamics (RQA/MdRQA1), average dyadic-level team dynamics (MdRQA2), and group-level dynamics (MdRQA3) as predictors in a simple regression analysis to predict the number of boats a team built, successfully and unsuccessfully, for each session individually. Figure 6 presents the results of the regression analysis in term of variance explained (*R*^2^) by each of the three group levels. In accordance with Håkonsson et al. (2015) and Mønster et al. (2016a), neither the individual level nor the dyadic level dynamics predicted well the number of boats built (*R*^2^ hovers around 0.1). In contrast, the analysis at the global group level showed a much stronger relation to the performance outcome, particularly in the later trials (*R*^2^ MdRQA3 increases to above 0.2 in Figure 7A). A strikingly similar picture is seen for the unsuccessful building attempts (Figure 7B). This suggests the existence of genuine group-level physiological processes in team interaction that span simultaneous interaction of all three group members and correlate with a key aspect of group performance but are neither located within the individual group members, nor in their dyadic interaction.

The current example illustrates how MdRQA can specifically be used in research of social interaction to systematically investigate (shared) dynamics at different group-levels. We identify a correlation between a global level physiological proxy for group arousal dynamics and an independent outcome measure of the team performance that could neither be seen at the level of individuals (Håkonsson et al., 2015) nor of dyads (Mønster et al., 2016a). This demonstrates the potential of MdRQA to explore different levels of aggregation within one analytical framework. Our finding could be interpreted as evidence for the presence of an interpersonal synergy (Riley et al., 2011) at the group-level, that is, interaction of all three team members is crucial for successful task performance, and this performance (or at least the emotional-arousal aspect of it) is not attributable solely to the individual group members, but emerges in their interaction.

It is likely that this type of dynamics depends on the specifics of the group interaction. In the present experiment, all group members were simultaneously present in the same room, working on the origami figures. However, there could be other group-setting, where only certain participants can interact with each other, or only interact with each other in certain ways that constrains their behavior (Wallot et al., 2016). We hypothesize that in this case dyadic interaction would more relevant for group performance, and hence we would see the strongest correlation with MdRQA2. In the same vein, we hypothesize that performance in automated assembly lines, where “social interaction” is fully—or primarily—determined by electronic control systems that are the pace-maker of the interaction, would be most informative at the individual level. We suggest that MdRQA provides a coherent analysis framework to test such hypotheses.

## A note on parameter estimation using MdRQA

Of course, a system with two (or more) measured variables could boast yet-higher dimensional dynamics than the two (or more) measured variables at hand. Then, it would be necessary to infer the appropriate dimensionality and reconstruct the phase-space by the method of time-delayed embedding (Takens, 1981). Here, one can start by assessing the delay and embedding parameters from the individual component signals that are eventually fed to MdRQA. For example, before running MdRQA on three signals (MdRQA3), one can test each signal's embedding parameters, and if dimensionality of the individual signals, as determined by a false-nearest-neighbor algorithm (Kennel et al., 1992) is, say, six, then the time-series consisting of three component signals could be embedded once to yield this dimensionality (i.e., 3 · 2 = 6). However, as these methods are just estimators for embedding parameters, one could also try to infer the delay and embedding parameters directly from the multidimensional signals (Clark et al., 2014).

Whether or not (or how) to embed cannot be answered conclusively by such estimation procedures, however. Embedding might not always be necessary. As March et al. (2005) showed, an unembedded recurrence plot—the “parent plot” (p. 194)—can, under given circumstances, contain all the information that embedded versions of this plot provide, and Iwanski and Bradley (1998) showed that recurrence variables for a variety of deterministic systems are invariant or at least highly similar over a range of embedding parameters, including the non-embedded versions. However, in our own practical experience analyzing behavioral and physiological data, considerations regarding the “adequate” embedding of the data does sometimes make a substantial difference for the results, and effects of embedding on the results should at least be investigated.

**Figure 8:**
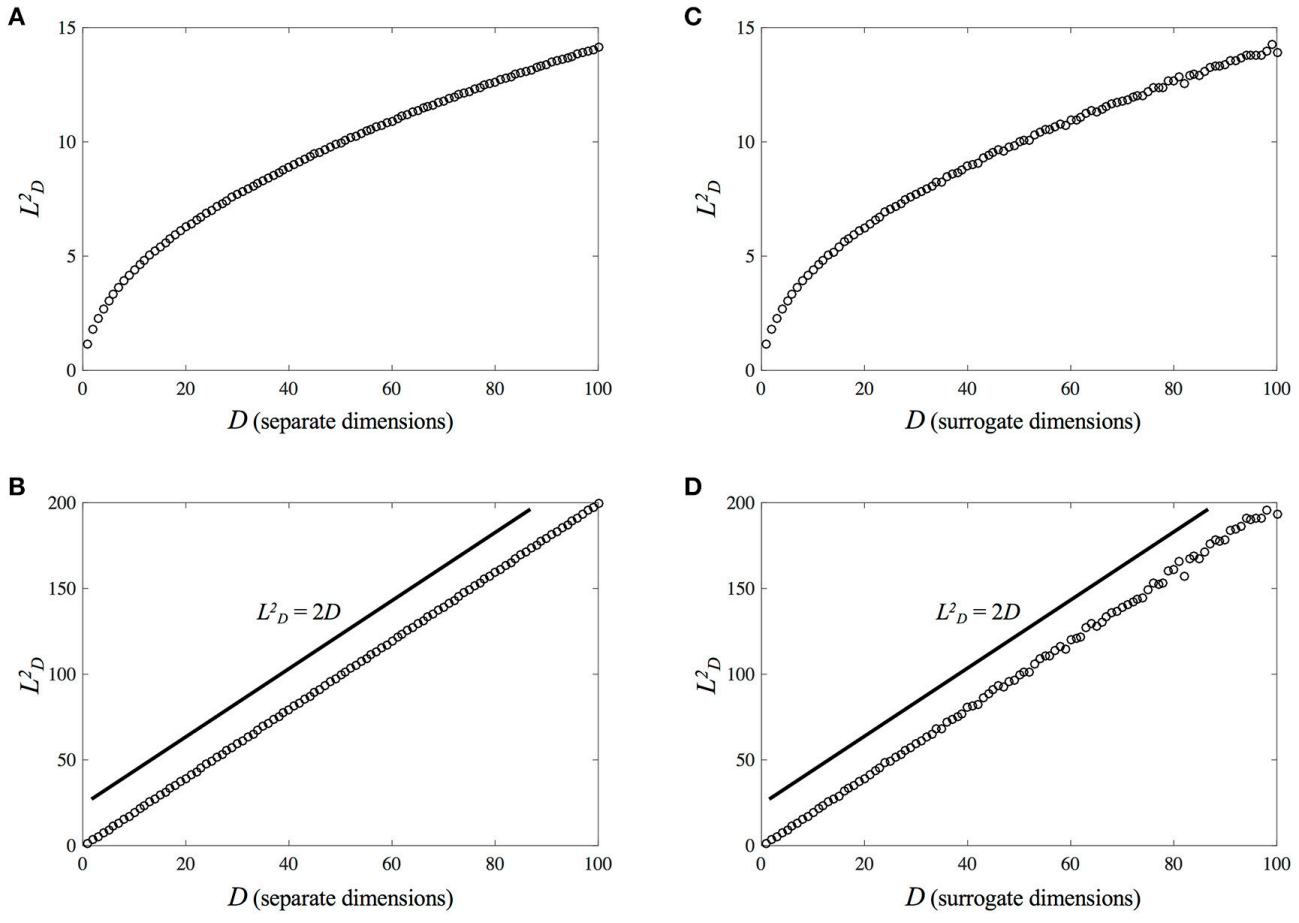
**Scaling of average phase-space distance with phase-space dimensionality (each dimension is a ***z***-scored random variable taken from a uniform distribution [0, 1]). (A)** Shows the increase of average distance as a function of separately added dimensions, and **(B)** shows that the increase in average distance follows the square-root of the dimensionality of the phase-space. **(C)** Shows the increase of average distance as a function of separately number of embeddings via time-delayed surrogates of a single random variable, and **(D)** shows that the increase in average distance follows the square-root of phase-space dimensionality as well. Distances in both cases scale similarly, with $L_{D}={\left(L_{D+n}^{2}\;-\;2n\right)}^{1/2}$.

Another issue is the question of comparing MdRPs of different dimensionality. If one is interested in comparing the magnitude of the different RQA-variables across a range of pairings of the component signals, using the analysis strategy we have described above [i.e., comparing for example DET for the individual signal (MdRQA1) vs. pairs of signals (MdRQA2) vs. the group-level (MdRQA3)], then one has to correct for the “baseline” effect of dimensionality on distances in phase-space and, subsequently, on all of the RQA outcome variables. Figure 8 illustrates this: Figures 8A,B shows how the average distance in phase-space increases as the square-root of subsequent dimensions added (each new dimension was a *z*-scored vector of random numbers drawn from a uniform distribution [0, 1]). This increase is similar to the increase in average phase-space distance when a single random variable is embedded in increasingly higher dimensions, see Figures 8C,D.

In particular, for random variables with equal variance, the average phase-space distance increases with dimensionality as $L_{D}^{2}=2D$, giving the scaling relation:

(11) $$\begin{array}{l} L_{D}=\sqrt{L_{D+n}^{2}-2n} \end{array}$$

where *L*_*D*_ is the average distance in phase-space given some dimensionality *D* of that space, and *L*_*D*+*n*_ is the average distance in a phase-space with *n* additional dimensions.

This can be taken as a baseline-correction factor to adjust the phase-space when one wants to compare RQA measures of, for example, a one-dimensional, non-embedded signal (RQA/MdRQA1) to three one-dimensional signals that are embedded together (i.e., MdRQA3). Alternatively, one could keep percent recurrence constant across RQAs obtained from phase-spaces with different dimensionality, and analyze other RQA measures, such as DET, ADL, or LDL. If, however, the one-dimensional signal is embedded in three dimensions using time-delayed surrogates, then such corrections are not necessary to compare RQA measures. This needs to be kept in mind if one wants to compare phase-spaces of different dimensionality using RQA/MdRQA, no matter whether the different dimensions are time-delayed surrogates or actual different observables.

## Interpretation of MdRQA, limitations, and potential future developments

As already mentioned in the last section, illustrating the application of MdRQA on skin-conductance measures during teamwork, as well as in the sections relating MdRQA to RQA, CRQA, and JRQA, there are two different, but related interpretations of MdRQA measures. On the one hand, we can interpret the outcome variables as capturing the dynamics of a (single) multidimensional system, as in the case of the Lorenz attractor, or as capturing synergistic relationship between different systems, as in the case of our skin-conductance example. Such interpretations might be more theoretically interesting, but could also put further demands on the data collected or explanations sought (i.e., is there a well-defined attractor manifold describing the dynamics of the variables? Can the coupling relationships between the variables be described in greater detail?). On the other hand, one can also simply view MdRQA as a tool to capture the simultaneous correlation of multiple variables over time—a form of dynamic multivariate correlation technique—that solves the problem of assessing multivariate correlation strength. In the former case, one would ideally investigate whether additional embedding is necessary (see consideration in the section “A note on parameter estimation in MdRQA”). In the latter case, one might consider simply using MdRQA on the non-embedded, one-dimensional component signals.

Besides the advantage of MdRQA, the ability to capture the dynamics of multiple signals at once, MdRQA also has disadvantages relative to other nonlinear coupling analyses, such as CRQA: At least with the method in its present form, it is not possible to calculate time-lagged coupling between signals to investigate leader-follower relationships among the component variables as with CRQA (Coco and Dale, 2014). It is also not possible to test the specific influence that one component signal has on another over time as with convergent cross-mapping (Mønster et al., 2016b). Solutions to this problem could be comparisons of different MdRPs with and without the specific signal of interest, such as in Joint Recurrence Analysis (Romano et al., 2004), or investigating the effects of time-shifting individual signals systematically and comparing the resulting MdRPs (as has been suggested by Marwan et al. (2007) for JRPs with two variables). Future developments in this direction would be desirable for a more accurate and detailed analysis of group-level performances beyond the dyad, and recurrence-based techniques seem very well suited to tackle such challenges.

## Author contributions

SW invented the method. SW and DM created the software. SW, DM, and AR were involved in collecting/creating the example data and wrote the article.

### Conflict of interest statement

The authors declare that the research was conducted in the absence of any commercial or financial relationships that could be construed as a potential conflict of interest.

## Footnotes

^1^For a full description we would also need the velocity as part of the phase-space, but we will ignore this here.
