# Supplementary material for: Multidimensional Recurrence Quantification Analysis (MdRQA) for the Analysis of Multidimensional Time-Series: A Software Implementation in MATLAB and Its Application to Group-Level Data in Joint Action
Source: Front Psychol. 2016 Nov 22;7:1835. doi: 10.3389/fpsyg.2016.01835 (PMC5118451; doi:10.3389/fpsyg.2016.01835)
Supplement: Supplementary file 1 [file DataSheet1.pdf]

Appendix: Definition of notation

Δt time interval

τ time delay

$\Theta(x)$ Heaviside step function

ADL average diagonal Line length

CRP cross-recurrence plot

CRQA cross-recurrence quantification analysis

D the (estimated) number of dimensions of a phase-space

d the number of variables used in a multivariate JRP

DET (%) percentage of deterministic recurrences

JRP joint recurrence plot

JRQA joint recurrence quantification analysis

LDL longest diagonal line length

L_D the phase space distance in D dimensions

MdRP multidimensional recurrence plot

MdRQA multidimensional recurrence quantification analysis

n the number of data points in a vector

N number of observables from which an MdRP is constructed

RP recurrence plot

RQA recurrence quantification analysis

RR (%) percentage of recurrence points

T threshold parameter

t a time point

$\mathbf{V}_i$ a D-dimensional vector of time delayed values of x

$\tilde{\mathbf{V}}_i$ a surrogate dimension of the reconstructed phase-space

$\mathbf{W}_i$ a D-dimensional vector of D observables y_1, $y_2, \ldots, y_D$ at time t_i

$\mathbf{x}$ a vector of values representing a one-dimensional time-series $(x_1, x_2, \ldots, x_n)$

$\mathbf{y}$ a vector of values representing a one-dimensional time-series $(y_1, y_2, \ldots, y_n)$

Appendix: MdRQA MATLAB Code

This appendix provides a MATLAB script to run MdRQA. The MATLAB code and the data used in examples of this article can be found under:

https://github.com/Wallot/MdRQA

```
function [RP, RESULTS,
PARAMETERS]=mdrqa(DATA,DIM,EMB,DEL,NORM,RAD,ZSCORE)
% mdrqa
%  computes a recurrence plot for a multi-dimensional time-series and
%  performs recurrence quantification:
%    [RP, RESULTS,
PARAMETERS]=mdrqa(DATA,DIM,EMB,DEL,RAD,NORM,ZSCORE)
%
%
% Inputs:
%
%  DATA is a double-variable with each dimension of the to-be-
analyzed
%  signal as a row of numbers in a separate column.
%
%  DIM is the dimensionality of the signal (i.e., the number of
colums that
%  should be read from the variable starting from the first).
%  The default value is DIM = 1.
%
%  EMB is the number of embedding dimensions (i.e., EMB = 1 would be
no
%  embedding via time-delayed surrogates, just using the provided
number of
%  colums as dimensions.
%  The default value is EMB = 1.
%
%  DEL is the delay parameter used for time-delayed embedding (if EMB
> 1).
%  The default value is DEL = 1.
%
```

```
%  NORM is the type of norm by with the phase-space is normalized.
The
%  following norms are available:
%    'euc' - Euclidean distance norm
%    'max' - Maximum distance norm
%    'min' - Minimum distance norm
%    'non' - no normalization of phase-space
%  The default value is NORM = 'euc'.
%
%  RAD is the threhold/radius size within points in phase-space are
counted
%  as being recurrent.
%  The default value is RAD = 1.
%
%  ZSCORE indicats, whether the data (i.e., the different columns of
DATA,
%  being the different signals or dimensions of a signal) should be
z-scored
%  before performing MdRQA:
%    0 - no z-scoring of DATA
%    1 - z-score columns of DATA
%  The default value is ZSCORE = 0.
%
%
% Outputs:
%
%  RP is a matrix holding the resulting recurrence plot.
%
%  RESULTS is a double-variable holding the following recurrence
variables:
%    1.  Size of the RP
%    2.  %REC  - percentage of recurrent points
%    3.  %DET  - percentage of diagonally adjacent recurrent points
%    4.  MeanL - average length of adjacent recurrent points
%    5.  MaxL  - maximum length of diagonally adjacent recurrent
points
%    6.  EntrL - Shannon entropy of distribution of diagonal lines
%    7.  %LAM  - percentage of vertically adjacent recurrent points
%    8.  MeanV - average length of diagonally adjacent recurrent
points
%    9.  MaxV  - maximum length of vertically adjacent recurrent
points
%    10. EntrV - Shannon entropy of distribution of vertical lines
%
%  PARAMETERS is a cell-variable holding the employed parameter
settings:
%    1. DIM
%    2. EMB
%    3. DEL
%    4. RAD
%    5. NORM
%    6. ZSCORE
%
```

```
% Reference:
%
%  Wallot, S., Roepstorff, A., & Mønster, D. (2016). Multidimensional
Recurrence Quantification Analysis (MdRQA) for the analysis of
multidimensional time-series: A software implementation in MATLAB and
its application to group-level data in joint action. Frontiers in
Psychology, ???

% Version:
%
% v1.0, 28. July 2016
% by Sebastian Wallot, Max Planck Insitute for Empirical Aesthetics,
Frankfurt, Germany
%  & Dan Mønster, Aarhus University, Aarhus, Denmark

if exist('DATA') % check whether input data has been specified - if
not, throw error message
else
    error('No input data specified.');
end

if exist('DIM') % check whether DIM has been specified - if not, take
maxium number of cloumns from DATA
    DATA=DATA(:,1:DIM);
else
    DIM_size=size(DIM);
end

if exist('EMB') % check whether EMB has been specified - if not, set
EMB = 1 (no surrogate embedding)
else
    EMB=1;
end

if exist('DEL') % check whether DEL has been specified - if not, set
DEL = 1 (no delaying)
else
    DEL=1;
end

if exist('NORM')  % check whether NORM has been specified - if not,
set NORM = 'euc'; if yes, check whether specification is appropriate
    if NORM == 'euc' | NORM == 'min' | NORM == 'max'
    else
        error('No appropriate norm parameter specified.');
    end
else
    NORM == 'euc';
end

if exist('RAD') % check whether RAD has been specified - if not, set
arbitarily RAD = 1
else
    RAD=1;
end
```

```
if exist('ZSCORE') % check whether ZSCORE has been specified - if
not, don't zscore
    if ZSCORE == 0
    else
    DATA=zscore(DATA);
    end
else
end

if EMB > 1 % if EMB > 1, perform time-delayed embbedding
    for i = 1:EMB
        tempDATA(1:length(DATA)-(EMB-1)*DEL,1+DIM*(i-
1):DIM*i)=DATA(1+(i-1)*DEL:length(DATA)-(EMB-i)*DEL,:);
    end
    DATA=tempDATA;
    clear tempDATA
else
end

PARAMETERS={DIM,EMB,DEL,RAD,NORM,ZSCORE}; % store parameters

a=pdist2(DATA,DATA); % create distance matrix and recurrence matirx
a=abs(a)*-1;
if NORM == 'euc'
    b=mean(a(a<0));
    a=a/abs(b);
elseif NORM == 'min'
    b=max(a(a<0));
    a=a/abs(b);
elseif NORM == 'max'
    b=min(a(a<0));
    a=a/abs(b);
elseif NORM == 'non'
else
end
a=a+RAD;
a(a >= 0) = 1;
a(a < 0) = 0;
diag_hist=[];
vertical_hist=[];
for i = -(length(DATA)-1):length(DATA)-1 % caluculate diagonal line
distribution
    c=diag(a,i);
    d=bwlabel(c,8);
    d=tabulate(d);
    if d(1,1)==0
        d=d(2:end,2);
    else
```

```
        d=d(2);
    end
    diag_hist(length(diag_hist)+1:length(diag_hist)+length(d))=d;
end
diag_hist=diag_hist(diag_hist<max(diag_hist));
if isempty(diag_hist)
    diag_hist=0;
else
end

for i=1:length(DATA) % calculate vertical line distribution
    c=(a(:,i));
    v=bwlabel(c,8);
    v=tabulate(v);
    if v(1,1)==0
        v=v(2:end,2);
    else
        v=v(2);
    end

vertical_hist(length(vertical_hist)+1:length(vertical_hist)+length(v)
)=v;
end

RESULTS(1,1)=length(a); % calculate recurrence variables
RESULTS(1,2)=100*(sum(sum(a))-length(a))/(length(DATA)^2);
if RESULTS(1,2) > 0
RESULTS(1,3)=100*sum(diag_hist(diag_hist>1))/sum(diag_hist);
RESULTS(1,4)=mean(diag_hist(diag_hist>1));
RESULTS(1,5)=max(diag_hist);
[count,bin]=hist(diag_hist(diag_hist>1),min(diag_hist(diag_hist>1)):m
ax(diag_hist));
total=sum(count);
p=count./total;
del=find(count==0); p(del)=[];
RESULTS(1,6)=-1*sum(p.*log2(p));
RESULTS(1,7)=100*sum(vertical_hist(vertical_hist>1))/sum(vertical_his
t);
RESULTS(1,8)=mean(vertical_hist(vertical_hist>1));
RESULTS(1,9)=max(vertical_hist);
[count,bin]=hist(vertical_hist(vertical_hist>1),min(vertical_hist(ver
tical_hist>1)):max(vertical_hist));
total=sum(count);
p=count./total;
del=find(count==0); p(del)=[];
RESULTS(1,10)=-1*sum(p.*log2(p));
else
    RESULTS(1,3:10)=NaN;
end

RP=imrotate(a,90); % format recurrence plot
end
```
